# A novel weight suppression score associates with distinct eating disorder and ultra-processed food addiction symptoms compared to the traditional weight suppression measure among adults seeking outpatient nutrition counseling

**DOI:** 10.1186/s40337-024-01029-5

**Published:** 2024-06-09

**Authors:** David A. Wiss, Erica M. LaFata, A. Janet Tomiyama

**Affiliations:** 1 Department of Community Health Sciences, Fielding School of Public Health, University of California Los Angeles, 650 Young Drive South, Los Angeles, CA 90095 USA; 2 Drexel University Center for Weight Eating and Lifestyle Science, 3201 Chestnut Street, Philadelphia, PA 19104 USA; 3 Department of Psychology, University of California Los Angeles, 502 Portola Plaza, Los Angeles, CA 90095 USA

**Keywords:** Weight suppression, Eating disorders, Ultra-processed foods, Food addiction, Nutrition counseling

## Abstract

**Background:**

Weight suppression has been defined as diet-induced weight loss, traditionally operationalized as the difference between one’s highest and current weight. This concept has been studied in the context of eating disorders, but its value in predicting treatment outcomes has been inconsistent, which may be partially attributed to its calculation.

**Method:**

The current study operationalizes a novel weight suppression score, reflecting the midpoint between the lowest and highest adult weights among adults (N = 287, ages 21–75, 73.9% women) seeking outpatient treatment for disordered eating. This report compared the traditional weight suppression calculation to the novel weight suppression score in a simulated dataset to model their differential distributions. Next, we analyzed shared and distinct clinical correlates of traditional weight suppression versus the novel weight suppression score using clinical intake data.

**Results:**

The novel weight suppression score was significantly associated with meeting criteria for both eating disorders and ultra-processed food addiction and was more sensitive to detecting clinically relevant eating disorder symptomatology. However, the novel weight suppression score (vs. traditional weight suppression) was associated with fewer ultra-processed food addiction symptoms.

**Conclusion:**

The novel weight suppression score may be particularly relevant for those with eating disorders and ultra-processed food addiction, with more relevance to individual eating disorder compared to ultra-processed food addiction symptoms. Consideration of the novel weight suppression score in future research on eating behaviors should extend beyond just those with diagnosed eating disorders.

## Abstract (highlights)

Weight suppression has been associated with elevated eating disorder symptoms, traditionally measured by subtracting one’s current weight from one's highest weight. Our novel weight suppression score relates current weight to one’s adult midpoint and thus might be a more sensitive indicator of clinically relevant weight suppression. Compared to the traditional approach, this measurement approach was associated with a higher number of eating disorder symptoms and a lower number of ultra-processed food addiction symptoms. Future research using this novel approach might better identify those at risk for disordered eating as well as those who may have achieved recovery from ultra-processed food addiction.

## Background

It is commonly accepted that highly restrictive dieting is one cause of eating disorders (ED) [1–4]. Thoughts and/or behaviors aimed at restricting food intake, often toward the goal of changing one’s shape or weight, are referred to as dietary restraint and have been part of comprehensive assessments for abnormal eating [5]. Early work identified three important factors to consider when evaluating dietary restraint: (1) one’s history/frequency of dieting and overeating, (2) current dieting behavior, and (3) weight suppression (WS) [6]. This work defined WS as “significant diet-induced weight loss” sustained over time, which is how it is frequently conceptualized today. This construct has been utilized in ED treatment research based on the observation that individuals with high levels of dietary restraint often pursue the maintenance of weights below self-imposed thresholds to the detriment of their mental health.

For this reason, most investigators and clinicians define goal weights in treatment based on the weight reached before the onset of the ED [7]. The most utilized method of calculating WS is the difference between the highest (excluding pregnancy) and lowest adult weight. One assumption of this underweight-centric ED model is that all weight loss is pathological. Many clinicians impose this same assumption on other forms of disordered eating more common among individuals living in larger bodies, such as binge eating disorder (BED). In other words, any current weight less than one’s lifetime high would be considered weight suppressed, regardless of other relevant contexts such as historical weight trajectory or related comorbidities (such as substance use disorder). This broad definition of WS has led to interest in more specific ways to operationalize this construct [8, 9].

The unique utility of WS in ED research has been demonstrated in a large sample of young women with body image concerns. Specifically, the traditional WS calculation was associated with increased odds of future onset of anorexia nervosa (AN), bulimia nervosa (BN), and purging disorder, but not BED [10]. Importantly, WS did not correlate with dietary restraint, body mass index (BMI), thin-ideal internalization, body dissatisfaction, or negative affect, suggesting that WS seems to be a distinct construct from other established ED risk factors. While WS was associated with increased odds of future onset of AN, BN, and purging disorder, dietary restraint showed stronger associations with these outcomes than WS [10]. Taken together, WS may capture certain physiological facets of EDs (e.g., undernourishment), whereas dietary restraint captures psychological factors (e.g., obsessional thinking and compulsive behaviors), and both seem to be uniquely related to future ED diagnosis. An absence of findings linking WS to future onset of BED likely relates to differences in the weight trajectories and diagnostic indicators (e.g., absence of compensatory behaviors) among those with BED versus AN/BN.

Most of the existing research using the traditional approach for calculating WS has related primarily to WS predicting ED treatment outcomes (e.g., weight gain, abstinence from ED behaviors) and has yielded inconsistent findings [8]. A systematic review from 2018 of 12 studies found that the traditional measure of WS reliably predicted weight gain post-treatment among those with EDs (predominantly underweight or non-underweight samples) [9]. Such findings are unsurprising since WS produces psychobiological pressures toward weight regain, including reduced metabolic rate and increased appetite [11]. It has been suggested that much of the biological opposition to WS is mediated by the adipocyte-derived hormone leptin [12, 13]. Research among women with BN has found significant associations between greater WS, lower leptin levels, and longer duration of illness, with leptin mediating the relationship between WS and illness duration [14]. Changes in the affective response and the rewarding properties of highly palatable food likely contribute to a decreased ability to engage in dietary restraint, thereby increasing loss-of-control food consumption [13]. Further, diminished leptin and glucagon-like peptide 1 (GLP-1) may contribute to alterations in reward valuation (i.e., increased salience) and the associated increased motivation to achieve satiation (both homeostatic and hedonic) [11]. In a randomized controlled prevention trial (focused on small sustainable diet and exercise behaviors to prevent weight gain among those without ED diagnoses [15]), those with the highest WS and lowest baseline BMI gained weight the most rapidly over two years post-intervention [16].

Yet, other studies of individuals in ED treatments have not observed associations between the traditional approach of calculating WS and treatment outcomes. In two separate studies of mixed adults with BN or BED, WS did not significantly predict treatment completion, weight change during treatment, or abstinence from binge eating [17]. Furthermore, in a sample of female outpatients ages 16–54 with bulimic disorders, WS was not significantly associated with treatment dropout or non-abstinence [18]. These authors recommended using more specific definitions of WS in future research. While some have recommended exploring WS as a moderator of ED treatment outcomes [9], the traditional WS measure has failed to moderate the effects of prevention programs [16].

Importantly, some researchers have partially attributed these inconsistent findings to limitations in the traditional approach to calculating WS [17]. For example, a 22-year-old female with AN who is 5′ 8″, currently at her lifetime adult lowest weight of 100 lbs. and 120 lbs. at her highest, would be assigned the same weight suppression score (i.e., 20) as a 42-year-old male with BN who is 5′ 8″, currently at his lifetime adult lowest weight of 200 lbs. and 220 lbs. at his highest (persons A & E in Table 1).

**Table 1:**
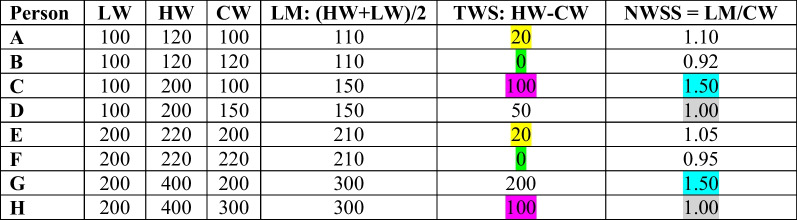
Hypothetical weights for two different weight suppression measurement methods

Matching colors indicate matching values to highlight discrepancies across the two approaches

*LW* Lowest adult weight; *HW* Highest adult weight; *CW* Current weight; *LM* Lifetime adult midpoint, *TWS* Traditional weight suppression; *NWSS* Novel weight suppression score

Thus, efforts to measure WS more effectively have led some investigators to calculate WS by subtracting the current BMI from the highest BMI [19–21] to account for changes in the relationship between height and weight. Using this approach, one study observed that WS was associated with increased symptoms of ultra-processed food addiction (UPFA), measured by the Yale Food Addiction Scale 2.0, among controls without EDs but not individuals with BN [19]. While substantial comorbidities between UPFA and EDs make it difficult to discern clinically relevant symptomatology [5, 22], these findings suggest that WS-related factors may also be relevant for individuals with UPFA and without other EDs, but the directionality remains unclear.

Furthermore, a recent review on WS in relation to ED and weight outcomes recommended developmentally sensitive calculations of WS [8]. Such efforts to advance the concept of BMI-based WS have utilized BMI z-scores (based on expected weight for height), which are particularly important for investigating correlates of EDs among youth [23]. The authors have referred to this as “developmental weight suppression” since it accounts for the age at which BMIs were reached. Among adult females with BN, this approach indicated more consistent relationships with bulimic characteristics (e.g., bingeing, purging) than the traditional WS calculation [24]. However, while the BMI z-score-based calculation of WS seems useful for individuals before 21 years old, it may be less applicable for those experiencing weight fluctuations beyond developmental years.

Several other methods have been proposed to refine the approach for calculating WS, including relative WS (the percentage of total body weight loss from the highest past weight) [25] and clarifying the intentionality of the weight loss leading to WS [26]. Clearly, there is empirical interest in developing more precise measures of WS that may be used as predictors, mediators, moderators, or outcomes in eating behavior research. One approach not documented in the scientific literature is the clinical practice that considers one’s current weight and involves comparing the current weight to the midpoint (average) between the lowest and highest adult weights. This approach has been used in some clinical settings as the preferred method for determining realistic weight goals for individuals seeking nutrition counseling. Specifically, if a patient has a goal weight less than their low/high midpoint, ED-informed professionals might view this goal as unrealistic/disordered and provide psychoeducation about the long-term stability of weight and the associated harms of weight cycling [27, 28].

Thus, this report aims to compare the traditional WS (TWS) calculation to this novel weight suppression score (NWSS) in a simulated dataset to model their differential distributions and consider how they might be operationalized in statistical models. Next, we examine the shared and distinct clinical correlates of the TWS versus the NWSS approaches using cross-sectional data from a private nutrition counseling practice. We hypothesized that the more sensitive NWSS would correlate with more ED symptoms than the TWS.

## Methods

### Simulation data for novel weight suppression score (NWSS)

A random set of 1,566 observations was created using Stata 18 [29] (Stata code is in *italics* below for easy replication). This number was adjusted post-hoc to create a sample size matching our observational data. Given that the average weight of adults (ages 20+) in the US is approximately 185 pounds [30], three separate Poisson distributions (N = 1566 each) with means approximately equal to 185 were created. A brief discussion accompanies the methods for the simulation data, whereas the results and discussion section below are reserved for the patient dataset. The simulation was conducted prior to the analysis of the observational data to conceptualize the difference between the two approaches. The objectives were to (1) propose a novel weight suppression score (based on clinical experience) rather than a crude difference measure, (2) visualize the distribution of the two approaches, and (3) create a variable that could be used in future research either as a moderator or outcome (rather than just a predictor) in regression models.

- *set seed 5249*
- *set obs 1566*

- *gen e1 *= *rpoisson(185)*
- *gen e2 *= *rpoisson(185)*
- *gen e3 *= *rpoisson(185)*

The first distribution was determined as the lowest adult weight (LW), the second distribution as the highest adult weight (HW), and the third as the current weight (CW).

- *gen LW *= *e1*
- *gen HW *= *e2*
- *gen CW *= *e3*

A dataset was then created by removing all implausible values: simulated observations were kept only if the lowest adult weight was less than the highest adult weight and if the current weight was less than or equal to the highest weight and greater than or equal to the lowest weight. Because this simulation data is random, the final values are unlikely to reflect what is reported in the observational data; they are offered to show how the NWSS reflects a normally distributed variable that can be dichotomized for analyses.

- *keep if LW* < *HW*
- *keep if CW *≤ *HW & CW *≥ *LW*

The final dataset for analysis contained 287 observations.

### Traditional weight suppression (TWS) method

**Fig. 1:**
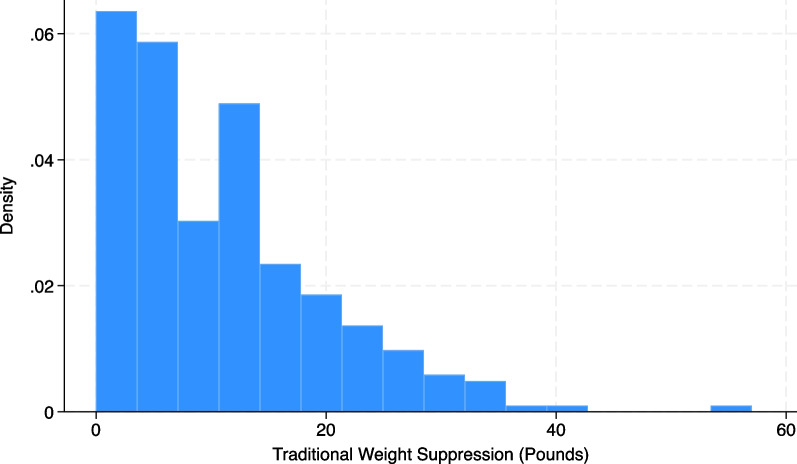
Distribution of traditional weight suppression using simulated data (N = 287)

The traditional weight suppression (TWS) approach subtracts the highest adult weight from the current weight [9], shown in Fig. 1.

- *gen TWS* = *(HW − CW)*
- *hist TWS*

As expected, this is not normally distributed (median < mean). This variable may be used as a linear predictor, but the approach poses methodological challenges for dichotomizing the variable for indicator analysis.

Importantly, this method is also flawed because it would assign the same value to someone with a lifetime high of 200 lbs. who is currently 100 lbs. (reduced weight by 50%) as it would to someone with a lifetime high of 400 lbs. who is currently 300 lbs. (reduced weight by 25%) (see persons C and H in Table 1, color-coded to indicate the same values). In addition, this approach lacks a nuanced context of weight history.

### Novel weight suppression score (NWSS) method

**Fig. 2:**
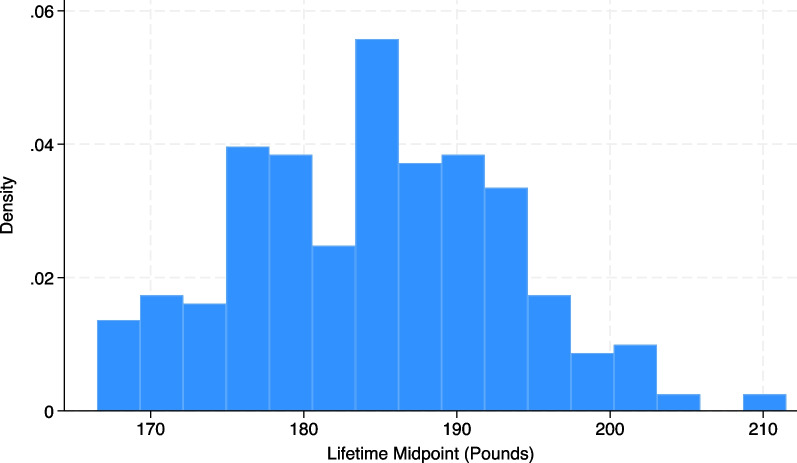
Distribution of lifetime midpoint using simulated data (N = 287)

Next, a lifetime (adult) midpoint variable (LM) was created by averaging the lowest adult weight (LW) and the highest adult weight (HW), as shown in Fig. 2.

- *gen LM* = *(LW* + *HW)/2*
- *hist LM*

**Fig. 3:**
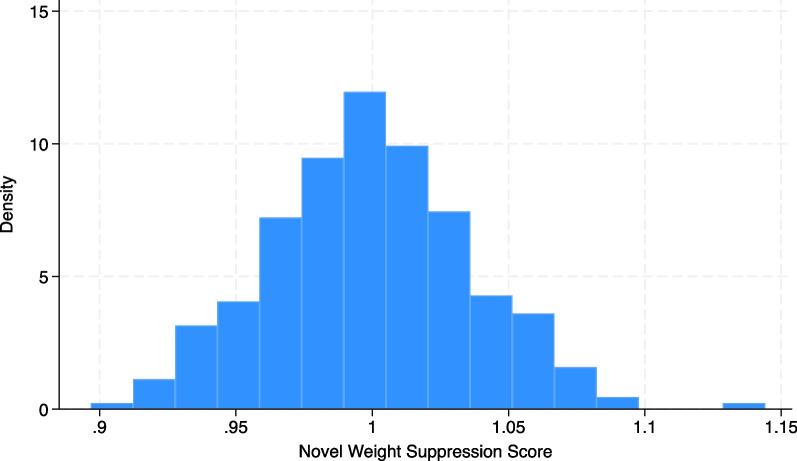
Distribution of novel weight suppression score using simulated data (N = 287)

As expected, this is normally distributed (the median is approximately equal to the mean). Next, we generate the NWSS by dividing the lifetime (adult) midpoint (LM) by the current weight (CW), creating more stringent criteria for suppression than the TWS approach (Fig. 3).

- *gen NWSS* = *LM/CW*
- *hist NWSS*

As expected, this is again normally distributed (the median is approximately equal to the mean), which may lend itself to use as a linear outcome or a dichotomized variable for indicator analysis. Conceptually, this captures a more notable phenomenon of weight suppression, which might be associated with a different clinical picture (which we put to the test below). Using this novel method, values above the mean could represent a status of weight suppressed. Technically, values above one could represent the weight-suppressed group using this definition, but we use the mean for consistency in comparing the two approaches. Many values below the mean would be considered weight suppressed using the TWS approach, warranting clarification on the most clinically relevant classification, potentially explaining inconsistent findings in previous research. Table 1 illustrates the differences in the WS values that would be generated by the TWS versus NWSS methods for eight hypothetical case examples (chosen to compare the two measurement approaches, which are on different scales). Matching colors indicate matching scores for numerous case examples using each WS calculation.

Importantly, the values assigned by TWS do not discern between the person who lost 50% of their body weight (person C) and the person who lost 25% of their body weight (person H). Such differences might be registered by the brain/body differently, where relatively higher percentages may be associated with more ED symptoms, but this needs to be empirically tested by holding the highest weight constant. In contrast, NWSS more appropriately considers the larger context of one's weight history (adult weight range), which makes it a more sensitive indicator likely to capture different clinical characteristics (e.g., assigning a much higher WS value to person C versus person H). The following analysis compares the associations of these two WS approaches in a clinical sample.

### Intake data from private nutrition counseling practice

Data collected come from a HIPAA-compliant online intake process (single time point) at a private (cash-based) group nutrition counseling practice in Los Angeles, CA. Clinicians are registered dietitian nutritionists specializing in the nutritional management of eating and substance use disorders, but patients sought counseling for a wide range of reasons. The study was approved by the University of California Los Angeles Institutional Review Board (IRB# 20-008829). Subjects were eligible for enrollment if they consented to participate in the study (20.7% of the potential sample opted out). Because the current study asked about one’s highest and lowest adult weights, analysis was restricted to those ages 21 and above. Study enrollment began in September 2020 and ended in April 2024. Final analysis occurred in April 2024.

### Sample characteristics

Demographic characteristics of the study sample are described in Table 2 (separated by gender), with additional columns for those screening positive for EDs, as operationalized by the short version of the Eating Disorder Examination Questionnaire (EDE-QS), and those meeting the criteria for UPFA, as operationalized by the modified Yale Food Addiction Scale version 2.0 (mYFAS 2.0). In the study sample, 177/287 (61.7%) screened positive for an ED (mean EDE-QS score = 16.2, SD = 8.5), 116/287 (40.4%) met the criteria for either moderate or severe UPFA (mean UPFA score = 3.6; SD = 3.5), and 104/272 (36.2%) met the established thresholds for both simultaneously. Of the 116 individuals who met the criteria for UPFA, 10.3% (n = 12) did not screen positive for an ED. Further, of the 171 persons who screened positive for an ED, 42.7% (n = 73) did not meet the criteria for UPFA. 98/287 (34.1%) did not meet thresholds for either ED or UPFA. Thus, the overlap between UPFA and EDs observed in this sample was comparable to prior studies in clinical settings [31]. The only gender difference that emerged was the association between TWS and screening positive for ED and UPFA, which was observed among women but not non-women. However, the NWSS did detect these associations among men. Further, given that 55.7% of the sample reported a BMI of $\ge$ 25 kg/m^2^, differences in the associations of NWSS versus TWS with symptoms of disordered eating and UPFA were explored for individuals with BMI < versus $\ge$ 25 kg/m^2^. Overall, similar conclusions were derived from each BMI subgroup as with the entire sample, and dichotomizing by BMI resulted in small cell sizes that prevented the ability to conduct all analyses using that approach. Thus, for simplicity and appropriate power, results are reported for the whole sample.

**Table 2:** Demographic characteristics of study sample by eating disorder and ultra-processed food addiction positive screens (N = 287)

	Women (n = 212)			Not women (n = 75)		
Characteristic	n (%) (n = 212)	EDE-QS+ (n = 135)	UPFA+ (n = 88)	n (%) (n = 75)	EDE-QS+ (n = 42)	UPFA+ (n = 28)
Age (Years)						
21–29	55 (25.9)	36 (26.7)	22 (25.0)	19 (25.3)	15 (35.7)	11 (39.3)
30–39	65 (30.7)	41 (30.4)	21 (23.9)	21 (28.0)	11 (26.2)	8 (28.6)
40–49	33 (15.6)	19 (14.1)	17 (19.3)	14 (18.7)	5 (11.9)	5 (17.9)
50+	59 (27.8)	39 (28.9)	28 (31.8)	21 (28.0)	11 (26.2)	4 (14.3)
Race/Ethnicity						
Not white	173 (81.6)	109 (80.7)	72 (81.8)	63 (84.0)	32 (76.2)	23 (82.1)
White	39 (18.4)	26 (19.3)	16 (18.2)	12 (16.0)	10 (23.8)	5 (17.9)
Education						
HS or less	16 (7.6)	12 (8.9)	8 (9.1)	9 (12.0)	5 (11.9)	2 (7.1)
Some college	49 (23.1)	30 (22.2)	16 (18.2)	24 (32.0)	12 (28.6)	9 (32.1)
College	80 (37.7)	52 (38.5)	30 (34.1)	30 (40.0)	21 (50.0)	13 (46.4)
Graduate school	67 (31.6)	41 (30.4)	34 (38.6)	12 (16.0)	4 (9.5)	3 (14.3)
Parental education						
Not college grad	61 (28.8)	39 (28.9)	22 (25.0)	22 (29.3)	9 (21.4)	7 (25.0)
College grad	151 (71.2)	96 (71.1)	66 (75.0)	53 (70.7)	33 (78.6)	21 (75.0)
BMI		*	*		*	*
Below 25	114 (53.8)	62 (45.9)	39 (44.3)	13 (17.3)	3 (7.1)	1 (3.6)
25+	98 (46.2)	73 (54.1)	49 (55.7)	62 (82.7)	39 (92.9)	27 (96.4)
Lifetime SUD						
No	101 (47.6)	61 (45.2)	37 (42.1)	23 (30.7)	13 (31.0)	8 (28.6)
Yes	111 (52.4)	74 (54.8)	51 (58.0)	52 (69.3)	29 (69.1)	20 (71.4)
Weight suppressed–TWS		*	*			
Below average	134 (63.2)	78 (57.8)	45 (51.1)	39 (52.0)	21 (50.0)	12 (42.9)
Above average	78 (36.8)	57 (42.2)	43 (48.9)	36 (48.0)	21 (50.0)	16 (57.1)
Weight suppressed–NWSS		*	*		*	*
Below average	102 (48.1)	72 (53.3)	50 (56.8)	49 (65.3)	34 (81.0)	24 (85.7)
Above average	110 (51.9)	63 (46.7)	38 (43.2)	26 (34.7)	8 (19.1)	4 (14.3)

*EDE-QS* Eating disorder examination questionnaire short; *UPFA* Ultra-processed food addiction, *HS* High school; *BMI* Body Mass Index; *SUD* Substance use disorder, *TWS* Traditional weight suppression; *NWSS* Novel weight suppression score

*Significant chi-squared test at *p* < 0.05 (tested for EDE-QS and UPFA)

### Measures

Standard demographic data on age, gender (non-binary collapsed into non-woman due to small cell size), race/ethnicity (non-Hispanic Black, Hispanic/Latin, Asian, Other/Mixed, Prefer Not to Say collapsed into non-White due to small cell sizes), education, parent’s level of education, self-reported current or previous alcohol/substance use disorder (yes/no), and self-reported height and weight, including lowest and highest (excluding pregnancy) adult weights was collected. Two participants reported lifetime highest weights that were less than lifetime lowest weights, and those observations were dropped. There were no missing data.

#### Initial symptom survey

This screening tool is used by Oxford Biomedical Technologies to assess eligibility for the Lifestyle Eating and Performance (LEAP) Mediator Release Test (https://www.nowleap.com/) by certified LEAP therapists. This screening tool has yet to be formally validated but is used by clinicians to assess the frequency of various somatic symptoms. Eighty symptoms are classified as never/rarely; mild/occasional; mild/frequent; severe/occasional; and severe/frequent. Indicator variables were classified as mild/occasional or less and compared to mild/frequent or more. Three variables: (1) food cravings; (2) binge eating or drinking; and (3) purging (all methods) were selected for analysis because they were hypothesized to correlate with the two WS measures compared in the current study (Table 3). Data generated from this instrument can be considered preliminary, as follow-up studies are needed. Notwithstanding, research using this symptom survey has been published [32].

**Table 3:** Chi-Squared analysis of associations between indicator variables with each weight suppression calculation, dichotomized at the mean (N = 287)

	TWS (n = 107)		NWSS (n = 129)	
Indicator variable	n (%) Among WS+	Chi-square	n (%) Among WS+	Chi-square
More likely to crave food more often (n = 203)	91 (44.8)	7.55**	82 (40.4)	13.60**
More likely to binge more often (n = 163)	80 (49.1)	13.80**	64 (39.3)	9.98**
More likely to purge more often (n = 34)	10 (29.4)	1.71	26 (76.5)	13.09**
More likely to perceive discrimination based on weight (n = 70)	44 (62.9)	20.70**	22 (31.4)	9.46**
More likely to consume UPFs more often (n = 133)	58 (43.6)	1.56	52 (39.1)	6.83**
More likely to meet criteria for ED (n = 177)	78 (44.1)	3.64	71 (40.1)	9.80**
More likely to meet criteria for UPFA (n = 116)	59 (50.9)	10.09**	42 (36.2)	9.76**

*TWS* Traditional weight suppression; *NWSS* Novel weight suppression score; *WS* Weight suppressed, *UPF* Ultra-processed foods; *ED* Eating disorder; *UPFA* Ultra-processed food addiction

*Significant at *p* < 0.05; **Significant at *p* < 0.01

#### Everyday discrimination scale

The Everyday Discrimination Scale was originally developed to detect experiences of racism encountered by African Americans [33] and has been validated for population health research on racism and health [34]. Questions ask about the frequency of perceived discrimination (from never to almost every day). Expanded versions of the measure ask about the reasons for these experiences. Given the explosion of research on weight stigma in recent years [35], listing weight as a reason for perceived discrimination has become increasingly common. Given that 24.4% of our study sample reported weight as a reason for experiencing discrimination, this variable was hypothesized to correlate with the two WS measures compared to the current study (Table 3).

#### Ultra-processed food consumption

A food frequency questionnaire (FFQ) developed by the private practice asked about the frequency of various foods consumed (0 = never, 1 = 1–3 times/month, 2 = 1–2 times/week, 3 = 3–4 times/week, 4 = 5–6 times/week, 5 = once/day, 6 = twice/day, 7 = three times/day, and 8 = four or more times/day). The NOVA classification [36] was used to classify ultra-processed foods (UPFs) based on the following: sugar-sweetened beverages, diet beverages, fried food, sweets/desserts, refined grains, alternative dairy products, and protein powders. An index was created combining the frequency of these foods, which created a normally distributed variable that was dichotomized at the mean to compare those with above-average UPF consumption to those below average (see Table 3). While this instrument is not validated, it was included as one minor exploratory variable to detect potential differences between WS measurement methods.

#### Eating disorder examination question short form (EDE-QS)

The widely used EDE-Q has a validated shortened version, which is a 12-item version with a 4-point response scale that asks about the frequency of behaviors over the last 7 days: (1) 0 days; (2) 1–2 days; (3) 3–5 days; (4) 6–7 days [37]. The total score ranges from 0 to 36, where scores at or above 15 are determined to be likely to have an ED (sensitivity = 0.83, specificity = 0.85) [38], which was used as an indicator variable (Table 3). For the individual item analysis, indicator variables were created for experiencing the symptom 3–7 days and compared to 0–2 days (Table 4).

**Table 4:** Chi-squared analysis of associations between EDE-QS items (< 2 Vs. 3–7 Days) with each weight suppression calculation, dichotomized at the mean (N = 287)

	TWS (n = 107)		NWSS (n = 129)	
EDE-QS Individual items	n (%) among WS+	Chi-square	n (%) among WS+	Chi-square
Deliberately trying to influence weight/shape (n = 165)	74 (44.9)	4.26*	73 (44.2)	1.54
Long periods of time without eating to influence weight/shape (n = 59)	29 (49.2)	2.76	28 (47.5)	0.00
Thoughts food/eating/calories make it difficult to concentrate (n = 91)	31 (34.1)	1.78	48 (52.8)	1.54
Thoughts weight/shape make it difficult to concentrate (n = 113)	49 (43.4)	1.03	44 (38.9)	5.33*
Definite fear that you might gain weight (n = 175)	77 (44.0)	3.43	73 (41.7)	5.79*
Strong desire to lose weight (n = 209)	99 (47.4)	18.78**	79 (37.8)	28.35**
Tried to control weight by vomiting or taking laxatives (n = 13)	4 (30.8)	0.46	11 (84.6)	7.57**
Compulsive exercise to control weight/shape (n = 52)	12 (23.1)	7.35**	33 (63.5)	6.58*
Sense of loss of control (n = 119)	54 (45.4)	2.72	46 (38.7)	6.22*
Eat what other people would regard as unusually large amount (n = 78)	40 (51.3)	5.98*	30 (38.5)	3.42
Weight/shape influenced how you judge yourself (n = 223)	101 (45.3)	12.96**	94 (42.2)	10.99**
Dissatisfied with weight/shape (n = 221)	102 (46.2)	16.61**	85 (38.5)	30.71**

*EDE-QS* Eating disorder examination questionnaire short; *TWS* Traditional weight suppression, *NWSS* Novel weight suppression score; *WS* Weight suppressed

*Significant at *p* < 0.05; **significant at *p* < 0.01

#### Modified Yale food addiction scale 2.0 (mYFAS 2.0)

The widely used Yale Food Addiction Scale has a validated shorted version, which is a 13-item questionnaire based on DSM-5 criteria for substance use disorder [39]. Two questions indicate clinical significance, and one must be present for a positive screen. Questions have different thresholds for meeting criteria and are then classified as no (0–1 symptoms or does not meet criteria for clinical significance), mild (2–3 symptoms), moderate (4–5 symptoms), or severe food addiction (6+ symptoms). An indicator variable was created for those with moderate/severe food addiction and compared to individuals with none/mild since this is a clinical population with high rates of eating and substance use disorders. For the individual item analysis, indicator variables were created based on the established thresholds for that item (Table 5).

**Table 5:** Chi-Squared analysis of associations between ultra-processed food addiction symptoms with each weight suppression calculation, dichotomized at the mean (N = 287)

mYFAS 2.0 individual items based on DSM-5 SUD criteria	TWS (n = 107)		NWSS (n = 129)	
	n (%) among WS+	Chi-square	n (%) among WS+	Chi-square
UPF taken in larger amount and for longer period than intended (n = 84)	38 (45.2)	1.51	34 (40.5)	2.27
Much time/activity to obtain, use, recover (n = 97)	47 (48.5)	4.67*	38 (39.2)	3.96*
Important social/occupational/recreational activities given up or reduced (n = 55)	26 (47.3)	1.62	25 (45.5)	0.10
Characteristic withdrawal symptoms; UPF taken to relieve withdrawal (n = 95)	47 (49.5)	5.64*	40 (42.1)	1.59
UPF use causes clinically significant distress (n = 171)	78 (45.6)	6.14*	64 (37.4)	16.83**
UPF use causes clinically significant impairment (n = 112)	58 (51.8)	11.17**	48 (42.9)	1.51
Failure to fulfill major role obligation (e.g., work, school, home) (n = 80)	41 (51.3)	6.16*	33 (41.3)	1.68
Use continues despite consequences (emotional/physical problems) (n = 151)	75 (49.7)	13.17**	55 (36.4)	15.36**
Tolerance (marked increase in amount; marked decrease in effect) (n = 102)	53 (52.0)	9.90**	38 (37.3)	6.52*
Craving, or a strong desire or urge to use (n = 121)	62 (51.2)	11.59**	46 (38.0)	7.37**
Persistent desire or repeated unsuccessful attempts to quit (n = 133)	68 (51.1)	13.47**	48 (36.1)	12.69**
Use in physically hazardous situations (n = 46)	23 (50.0)	2.42	24 (52.2)	0.50
Continued use despite social or interpersonal problems (n = 55)	32 (58.2)	9.68**	15 (27.3)	11.04**

*TWS* Traditional weight suppression; *NWSS* Novel weight suppression score; *WS* Weight suppressed; *DSM* Diagnostic and statistical manual; *SUD* Substance use disorder; *UPF* Ultra-processed food; *CS* Clinical significance

*Significant at *p* < 0.05; **significant at *p* < 0.01

## Results and discussion

Dichotomizing the TWS and NWSS at the mean to create indicator variables classified 39.7% as weight suppressed using the traditional approach, whereas 47.4% were classified as suppressed using the novel approach (results stratified by gender in Table 2). Table 3 indicates that 44.1% of those screening positive for ED were weight suppressed based on the TWS measure, whereas 40.1% were considered suppressed based on the NWSS. Following a similar pattern, 50.9% of those meeting the criteria for UPFA were weight-suppressed using the TWS, whereas 36.2% were classified as suppressed using the novel approach.

Next, we compared the prevalence of several indicator variables across the two measures of WS to report their chi-square test associations. Table 3 compares bivariate associations to relevant indicator variables using the two WS calculation methods, identifying their links to problematic eating symptomology.

The NWSS approach was associated with all the variables hypothesized to correlate with the WS construct. The TWS calculation was not associated with purging, likelihood of meeting criteria for ED, or above-average ultra-processed food (UPF) consumption frequency. As expected, the general trend was that the NWSS approach captured a smaller percentage of those positive for WS than the TWS. One exception was purging, endorsed at more than double the prevalence using the NWSS method. As the measure got more stringent (from TWS to NWSS), the association with purging increased, suggesting that this more sensitive measure of WS may be particularly useful in detecting the increased likelihood of specific ED behaviors. The increase in the number of statistically significant associations using the NWSS method (7/7 variables vs. 4/7 variables) indicates that it is a more sensitive measure than TWS and may be more appropriate for future research on pathological eating.

Table 4 parses out the individual items on the EDE-QS and compares bivariate associations to each item question using the different WS methods.

The same trend of decreasing prevalence in EDE-QS endorsement from TWS to NWSS was observed. Exceptions were questions 3) thoughts about food/eating/calories make it difficult to concentrate on interesting things; 7) purging or laxative use; and 8) compulsive exercise to control weight/shape or burn fat/calories. More individuals classified as weight suppressed using the NWSS report these symptoms more frequently, consistent with the theory that more suppression tracks along with ED symptoms. The TWS approach detects significant associations in 6/12 items, whereas the NWSS calculation detects significant associations in 8/12 items. Thus, the NWSS approach appears more sensitive to detecting clinically relevant ED symptomatology. Question 1) deliberately trying to influence weight/shape is one exception, where the significant correlation was lost when tested against the NWSS, suggesting that this more precise measurement method may be associated with less frequent effort to alter weight/shape.

Table 5 parses out the individual items (mapped onto its criteria for substance use disorder) on the mYFAS 2.0 and compares bivariate associations to each item question using the different weight suppression methods.

The same trend of decreasing prevalence in mYFAS 2.0 symptom endorsement from TWS to NWSS is observed. The one exception was criterion 12) use in physically hazardous situations. The TWS detected significant associations in 10/13 items, whereas the NWSS detected significant associations in 7/13 items. Loss of significant associations occurred in criteria 4) withdrawal symptoms; 6) clinically significant social impairment; and 7) failure to fill major role obligations. Thus, TWS appears to capture more associations between WS and UPFA symptoms than the NWSS. This may be because the NWSS calculation is more likely to categorize individuals as positive for WS if they have maintained weight loss, which has been associated with lower UPFA symptoms in prior research [40].

## Summary

Prior research has indicated a need for a more precise assessment of clinically relevant WS. Using simulation and cross-sectional data from a private dietitian practice, findings suggest that the NWSS approach is a more sensitive measure of WS and may be preferred when comparing a wider array of ED symptoms among those who do versus do not meet these novel criteria for WS. Importantly, compared to TWS, the NWSS calculation may be a more appropriate screening tool to determine one’s risk for exhibiting numerous symptoms of disordered eating based on their WS status. However, while the NWSS approach was associated with UPFA diagnosis, this calculation detected fewer significant associations than TWS between WS and individual symptoms of UPFA. Individuals who have maintained weight loss (which is better captured by the NWSS) may simultaneously experience reductions in UPFA, although longitudinal studies are needed to confirm this hypothesis. WS may be particularly relevant for those with EDs and those who meet the criteria for UPFA, with more relevance to individual ED symptoms compared to UPFA symptoms. This novel approach to measuring WS may help explain previous inconsistent findings among those with EDs. Given its more stringent criteria and normal distribution, the NWSS may be suitable for moderation analysis in statistical models.

### Limitations

Cross-sectional data cannot make inferences about the temporal sequence between symptoms of disordered eating and suppressed weight. The majority of published WS research has been conducted among those with diagnosed EDs, and study data did not include formal ED diagnoses. The sample contained 61.7% of individuals who screened positive for EDs, whereas the remaining study participants were community-based and not necessarily seeking treatment for disordered eating. Thus, findings cannot be extrapolated to ED-specific populations, as it remains important to separate ED recovery from weight loss. Importantly, over half of the participants reported a lifetime history of any substance use disorder, which makes this population unique. Additionally, factors such as medications that impact weight status were not accounted for, and analysis did not account for a history of bariatric surgery (although data came from a practice with a small number of bariatric patients based on clinician recall). Other limitations come from the need for more validation of the initial symptom survey and the FFQ, but these instruments were only used as a minor part of the exploratory analysis. FFQs are inconsistently reliable [41], as are self-reported weights [42]. Finally, simulation data was used only for conceptual purposes and could not generate any meaningful conclusions about the observational data. Despite these limitations, our analysis suggests that the NWSS may advance research on WS in the context of disordered eating.

## Conclusion and future directions

Our analysis proposed a novel approach for calculating WS that has the potential to account for a crude weight history. Preliminary evidence demonstrated that this new method of categorizing WS was more sensitive than the traditional WS calculation and was also associated with a wider array of ED symptomology. There are abundant opportunities for future research to use the NWSS calculation to advance empirical knowledge of the role of WS in EDs and UPFA. One key line of research will be the continued assessment of the utility of NWSS versus TWS (and other WS calculations) across various clinical populations (e.g., EDs, UPFA) by comparing its associations with clinically meaningful psychopathology (e.g., body image overconcern, internalized weight stigma), compulsive food intake in the context of reward sensitivity, and related metabolic markers (e.g., leptin). The NWSS might be conceptualized as a moderating variable when ED symptoms are the outcome. Incorporation of data on the consumption patterns of UPFs may be insightful. Longitudinal studies would be particularly impactful for advancing scientific understanding of the temporal relationships between WS, symptoms of EDs and UPFA, and changes in weight trajectories. Consideration of WS in future research on eating behaviors should extend beyond those with diagnosed EDs, also investigating those at risk, as well as those who may have achieved recovery from addiction-like eating.

## Data Availability

The data that support the findings of this study are available from the corresponding author upon reasonable request.

## Abbreviations

ED: Eating disorder
WS: Weight suppression
BED: Binge eating disorder
AN: Anorexia nervosa
BN: Bulimia nervosa
BMI: Body mass index
UPFA: Ultra-processed food addiction
TWS: Traditional weight suppression
NWSS: Novel weight suppression score
LW: Lowest weight
HW: Highest weight
CW: Current weight
LM: Lifetime Midpoint
EDE-QS: Eating disorder examination questionnaire short
FFQ: Food frequency questionnaire
UPF: Ultra-processed food
