# Correction: A novel weight suppression score associates with distinct eating disorder and ultra-processed food addiction symptoms compared to the traditional weight suppression measure among adults seeking outpatient nutrition counseling

**DOI:** 10.1186/s40337-024-01047-3

**Published:** 2024-06-19

**Authors:** David A. Wiss, Erica M. LaFata, A. Janet Tomiyama

**Affiliations:** 1 Department of Community Health Sciences, Fielding School of Public Health, University of California Los Angeles, 650 Young Drive South, Los Angeles, CA 90095 USA; 2 Drexel University Center for Weight Eating and Lifestyle Science, 3201 Chestnut Street, Philadelphia, PA 19104 USA; 3 Department of Psychology, University of California Los Angeles, 502 Portola Plaza, Los Angeles, CA 90095 USA

**Correction to: Wiss et al. Journal of Eating Disorders (2024) 12:75**

10.1186/s40337-024-01029-5.

Following the publication of the Original Article, the authors reported an error in the title. The word “addiction” after the word “food” was missing from the title:

**Incorrect:**

A novel weight suppression score associates with distinct eating disorder and ultra-processed food symptoms compared to the traditional weight suppression measure among adults seeking outpatient nutrition counseling.

**Correct:**

A novel weight suppression score associates with distinct eating disorder and ultra-processed food addiction symptoms compared to the traditional weight suppression measure among adults seeking outpatient nutrition counseling.

The title of this Correction article is correct, and the original article has been corrected.

